# Genomic information increases prediction accuracy of behavior traits of Labrador Retrievers used as guide dogs

**DOI:** 10.1186/s12711-026-01033-0

**Published:** 2026-03-01

**Authors:** Molly M. Riser, Jane Russenberger, Madeline Zimmermann, Frances L. Chen, Caroline Moeser, Eldin Leighton, Breno Fragomeni

**Affiliations:** 1 Department of Animal Science, University of Connecticut, Storrs, CT USA; 2 International Working Dog Registry, San Antonio, TX USA; 3 Canine Genetic Services, LLC, Clayton, NM USA; 4 Genomics and Computational Biology, UMass Chan Medical School, Worcester, MA USA; 5 Vertebrate Genomics, Broad Institute of MIT and Harvard, Cambridge, MA USA; 6 Guiding Dogs New South Wales/Australian Capital Territory, St Leonards, Australia; 7 Institute for System Genomics, University of Connecticut, Storrs, CT USA

## Abstract

**Background:**

This study aimed to evaluate the accuracy of prediction of breeding values in a genomic selection program for behavior traits in a population of Labrador Retrievers used as guide dogs. Implementing genomic selection as a new tool in service dogs has the potential to increase genetic gain, improving the performance of populations. Additionally, genomic predictions may help service dog organizations in identifying training candidates with higher accuracy.

**Results:**

Phenotypes for 17 traits on 4,841 Labrador Retrievers collected from 2008 to 2019 from the International Working Dog Registry’s (IWDR) behavior checklist were analyzed. The Behavior Checklist (BCL) standardizes a scoring system for a dog’s reaction to a variety of environmental stimuli. Data are used to assess a dog’s behavior and suitability for training as well as genetic selection using a selection index of prioritized traits with estimated breeding values. Genomic data were available for 1076 individuals from whole genome sequences and reduced to 94 K SNPs. Variance components were estimated using AIREML. Genomic information was included under a single-step GBLUP approach. Accuracies were evaluated among a sample of the higher accuracy animals using the linear regression method. Genomic estimates of heritability ranged from 0.08 to 0.21. Accuracies were calculated with the LR method and ranged from 0.30 to 0.58 for pedigree information, with an average of 0.46. Accuracies of genomic predictions ranged from 0.32 to 0.63, with an average of 0.50, and were higher than pedigree predictions for all traits.

**Conclusions:**

The gains in accuracy from inclusion of SNP genotype data show that genomic prediction using single-step GBLUP can improve selection by identifying the cohort of young dogs that have the highest genetic merit for the desired traits. Gains in validation accuracy were limited by the small number of genotyped animals and are expected to increase as more animals are genotyped.

## Background

According to the Americans with Disabilities Act of 1990, a service animal is any dog that is trained to do work or perform tasks for an individual with a disability, sometimes also called an assistance dog or working dog. An estimated 60% of dogs evaluated for assistance work graduate from training programs, resulting in an estimated loss of at least $12,182.95 per dismissed dog. In 2017, this inflated the total price of a placed working dog to between 20,000 and $50,000 [1]. However, recent prices listed on the Guiding Eyes [2] and Fidelco [3] web pages place this much closer to $50,000. Between 65 and 77% of dogs that are disqualified or fail training do so because of behavioral issues [4].

The complexity of canine behavior and the lack of a standardized scoring tool across populations have been a main challenge impeding effective analyses. Goddard et al. [5] and Wilsson et al. [6] studied the genetics of canine behavior associated with guide dogs’ success. However, the phenotypes proposed by those programs are limited the subjectivity of their measurements. Although the Behavior Checklist (BCL), as a standardized scoring tool coupled with a global scoring certification, is a major step forward, measures are still subjective [7]. Research efforts to obtain objective measures on aspects of behavior are promising [1, 8]. Genetic improvement to reduce the incidence of hip dysplasia [9] and retinal diseases [10] using estimated breeding values (EBVs) has been made within individual organizations [11]. However, the use of genotype data for genetic evaluation and selection of dogs bred for assistance work have fallen short when compared with livestock species. Recent work utilized genomic prediction to identify young dogs as replacement breeders to improve hip dysplasia [12]. Additionally, Friedrich et al. proposed an improved phenotypic assessment of canine behavior, including demographic and management factors [13].

Genomic selection programs have proven useful in making gains in performance in many agriculturally important species [14–20]. Genomic selection is commonly used in dairy and beef cattle to improve economically important traits [16–19, 21–27]. Canine industry leaders have suggested the utilization of genomic prediction in canine populations to assist with breeding decisions [28, 29]. In 2018, Leighton et al. proposed a breeding program protocol to identify and breed superior service dogs using genotype data and a sophisticated phenotypic collection proposed [29].

The extra genetic gain from genomic selection programs arises from higher accuracies of EBVs of young animals. Many factors affect the accuracy of genomic EBVs (GEBVs), such as the trait heritability, the number of individuals with phenotypes and genotypes, and the number of independent chromosome segments [30]. Additionally, the validation method used can affect the estimates of accuracies obtained and may influence selection of the best prediction model [31–33].

The high cost associated with low graduation rates indicates the need for an in-house breeding program for working dogs [29]. Such programs must aggregate data from multiple organizations that produce service dogs to achieve higher prediction accuracies. The International Working Dog Association (IWDA) and their International Working Dog Registry (IWDR) database assist in the breeding and selection of working dogs and help member organizations by managing data and providing tools to help select genetically more suitable dogs for breeding [34]. Therefore, the objective of this study was to assess the accuracy of genomic predictions and the feasibility of a genomic selection program for guide dogs using phenotypes from the IWDR behavior checklist, combined with low-pass whole genome sequences, by estimating genetic parameters and comparing the accuracy of pedigree-based and genomic EBVs.

## Methods

Institutional Animal Care and Use Committee (IACUC) approval was not required for this study because all data were obtained from an existing database and did not involve any new animal procedures or interventions.

### Phenotypic data

The phenotypic data used in this analysis represent single-record Behavior Checklist (BCL) that are collected from year to year by internationally accredited, nonprofit guide dog organizations, Guiding Eyes for the Blind (New York, USA) and Guide Dogs New South Wales (New South Wales, AUS). assigned by trained staff during a formal test prior to the dog starting professional training. A total of 52 BLC items representing behavior traits are collected, and 17 of those traits were used in the present study based on data availability and importance for guide dogs’ performance. A summarized description of the BCL scores is in Table 1. BCL data is routinely collected by IWDR subscribers, with phenotypes scored using a 5-point ordinal scale (1 = least desirable) for each trait [35]. The data collected from the BCL has been adopted as a reliable tool across working dog organizations and testing environments due to a standardized scoring system [36]. Evaluators are trained within their organizations with educational videos, webinars, and workshops provided by IWDA on how to assess these behaviors. Certification Testing for inter-rater agreement is also provided [7].

**Table 1:** Names, descriptions, and abbreviations of the behavior traits used in the prevailing figures and tables

Trait	Description	Abbreviations
Anxiety in New Situations	Anxious during first few visits to unfamiliar locations	AnxNewSit
Noise sensitivity	Startle, tense body language, escapes or displacement behaviors when exposed to loud noises excluding thunder.	Noise
Self-modulation	It takes the dog a long time to return to a productive emotional state following exposure to an arousing or stressful stimuli	Selfmod
Handle sensitivity	Drops rear quarters when harness handle lays on back	Hsens
Fear of under footings	Fearful or apprehensive of various walking surfaces	UF
Body sensitivity	Inhibited by physical contact with objects other than harness handle on back	Bsens
Activated by stress	Copes with stressful situations poorly evidenced by becoming more active with faster movements, taking treats harder, more distracted	Actstress
Inhibited by stress	Copes with stressful situations poorly evidenced passively by shutting down, avoidance and withdrawal as a response to stress	Inhibstress
Socially inappropriate with people	Exhibits poor social manners with people	SocInap
Distracted by scent	Distracted by olfactory stimuli	Sniff
Distracted by dogs	Persistent interest in and high excitability level with other dogs	Dogdist
Excited by movement	Easily distracted by non-animal movement and has trouble redirecting attention	Movexit
Excitable	Increases energy and arousal level without observed stress signals in response to stimuli	Excit
Novel objects	Fearful avoidant or suspicious of new or novel objects in the environment	NovelObj
Retreats from known person	Moves head or face away when reached for by known person	Retreats
Fear of strangers	Fearful, nervous, apprehensive of strangers	Strange
Body handling	Avoidant, anxious, or aggressive when being handled for noninvasive activities like a vet exam/grooming/nail trim	BHand

All the traits are scored on a scale of 1 (severe and least desirable) to 5 (absent and most desirable)

Phenotypic data were obtained from the IWDR BCL for 11,381 Labrador Retrievers for 17 traits. Those records were collected on a total of 3487 litters, between 1 and 12 animals per litter. There was no consistent prescreening or pre-selection of animals to be enrolled in the program. The litters were connected via pedigree information, with 1183 unique animals listed as sires, with between 1 and 116 phenotyped progeny, and 2042 dams with between 1 and 37 phenotyped progeny. After being pruned back to three generations, the pedigree file contained 13,913 animals born between 1991 and 2025. Phenotypic records were collected from 2008 to 2024, with the number of records per year varying from 313 to 1052, except for the first and last year of the dataset, which contained fewer than 100 records.

### Genotype data

Animals were chosen to be genotyped using a ranked system that prioritized individuals based on their number of progeny with phenotypes, followed by animals with their own phenotype records. Phenotypic performance did not influence the selection of animals to be enrolled in the genotyping list. Not all breeders were genotyped due to limited availability of biological samples. A total of 1111 dogs were genotyped, of which 631 had own records. Among the genotyped animals, 169 were sires with between 1 and 116 progeny with phenotypes, and 320 dams with between 1 and 37 progeny with phenotypes. A total of 220 animals with no behavior records or progeny were included in the analysis to assist with allele frequencies estimation and improve connectedness between records. Those animals were genotyped based on the availability of health records that were not used in this study.

Genomic information was initially available for 1113 animals based on low coverage (~ 1×) whole genome sequencing (WGS). Sequences were aligned to the canine reference genome (CanFam4) using BWA [37] and imputed to a haplotype reference panel using GLIMPSE [38] based on 676 sequenced dogs and 53 M sites. Imputed Variant Call Format (VCF) files were used to extract 142,759 SNPs using the software VCFtools [39]. SNPs were filtered from low-pass WGS data to retain only those present on the Embark custom high-density Illumina 220k SNP array (Embark Veterinary Inc., Boston, MA, USA), which contains 220,484 SNP covering all 38 autosomes [40]. SNPs not present in the VCF file and sex chromosomes were excluded. Additionally, SNPs with a gene content heritability lower than 0.975 were removed from the panel [41]. VCF files were converted to Browser Extensible Data (BED) format using the R package “gdsfmt” version 1.36.0 and “SNP Relate” version 1.34.1, using R version 4.2.2 [42, 43]. BED files were then transformed into an additive gene content matrix using Plink [44] version 1.90.

The SNP data were then subjected to a quality control process using preGSf90 [45]. A total of 48,538 SNPs with minor allele frequency (MAF) less than 0.05 were removed, including 15,001 monomorphic SNPs. No SNPs or animals presented a call rate less than 0.9. Additionally, 2 SNPs and 41 animals were removed due to Mendelian conflicts. Two animals that were not present in the pedigree file were also removed. After quality control processing, genotypes on a total of 94,219 SNPs were available for 1076 animals.

### Variance components estimation

Variance components for the 17 behavior traits were analyzed using a restricted maximum likelihood (AIREML) method [46] with the following the single-trait linear animal model:

1 $$\:\begin{array}{c}{y}_{ijkl}=se{x}_{i}+yo{b}_{j}+c{g}_{k}+{a}_{l}+{e}_{ijkl}\end{array}$$

where $\:{y}_{ijkl}$ is the behavior phenotype for animal (*a*) *l*, of sex *i*, born in year of birth (yob) *j*, and with data collected in contemporary group (cg) *k*, while $\:{e}_{ijkl}$ is the residual for this observation. Contemporary groups were defined by a concatenation of organization and calendar month and year that the dog was scored to more accurately compare animals that were tested under similar conditions. This attempts to mirror the herd/month/season contemporary group utilized in livestock genetic evaluations. There were 220 contemporary groups with between 4 and 58 dogs, with more than one evaluator per organization. Year of birth was included in the model as a categorical effect with 26 levels since the age of individuals varied within contemporary groups and year of birth provided a better fit than the fixed effect of age (results not shown). The effects of sex, cg, and yob were considered fixed, while *a* represents the random additive genetic effect, and e the residual associated with each observation. The assumptions for the random effects were:

2 $$\begin{array}{*{20}c} {\left[ {\begin{array}{*{20}c} \mathbf a \\ \mathbf e \\ \end{array} } \right] \sim N\left[ {\begin{array}{*{20}c} 0 \\ 0 \\ \end{array} } \right],\left[ {\begin{array}{*{20}c} {\mathbf A\sigma _{a}^{2} } & 0 \\ 0 & {\mathbf I\sigma _{e}^{2} } \\ \end{array} } \right],} \\ \end{array}$$

where ***A*** is the numerator relationship matrix, ***I*** is the identity matrix with rank equal to the number of animals, $\:{\sigma\:}_{a}^{2}$ is the additive genetic variance of the trait, and $\:{\sigma\:}_{e}^{2}$ is the residual variance. An approximation of the standard error (SE) of the estimates of variances and heritability was calculated as the standard deviation of the (co)variances or their functions by sampling the parameter estimates, which is an accurate approach to assess sampling variation and an alternative to Bayesian estimation using a Markov Chain Monte Carlo approach [47]. This method is implemented in the BLUPF90 + software that was used for the variance component analyses [48].

### Prediction of breeding values

Estimated breeding values (EBV) for each trait were calculated from a single-trait pedigree best linear unbiased prediction (BLUP) model using the AIREML variance components by solving the Henderson Mixed Model Equations (MME) system of equations [49]. Genomic information was incorporated to calculate GEBV for each traits under a single-step GBLUP (ssGBLUP) approach [21, 50]. This method allows inclusion of genotyped and non-genotyped animals in the same analysis by fitting a relationship matrix that includes full pedigree and partial genomic information (***H***). The inverse of this matrix is:

3 $$\:\begin{array}{c}{\boldsymbol{H}}^{-1}={\boldsymbol{A}}^{-1}+\left[\begin{array}{cc}0&\:0\\\:0&\:{\boldsymbol{G}}^{-1}-{\boldsymbol{A}}_{22}^{-1}\end{array}\right],\end{array}$$

where ***A***^**− 1**^ is the inverse of the numerator relationship matrix, ***A***_**22**_^**−1**^ is the inverse of the partition of ***A*** that includes genotyped animals, ***G***^**− 1**^ is the inverse of the genomic relationship matrix given by:

4 $$\:\begin{array}{c}G=\frac{{\boldsymbol{M}}^{\boldsymbol{{\prime\:}}}\boldsymbol{D}\boldsymbol{M}}{{\sum\:}_{j=1}^{m}2{p}_{j}\left(1-{p}_{j}\right)},\end{array}$$

where ***M*** is a centered matrix of gene content based on *m* SNP markers, ***D*** a diagonal matrix of SNP weights, and $\:{p}_{j}$ is the major allele frequency for SNP *j*.

### Validation

The accuracy of EBV was assessed using the linear regression (LR) method, proposed by Legarra and Reverter [32]. In the first step of the LR method, GEBVs were calculated using the complete data (GEBV_COM_). The GEBV_COM_ provided the benchmark for EBV and GEBV computed on validation animals in the second step, in which 100 individuals were selected randomly to be included in the validation group. Only genotyped animals with their own phenotypes and at least ten progeny could be included in the validation group. A total of 186 out of the 1,076 genotyped animals qualified to be included in the validation set. This approach resulted in validation animals being selected across many generations, which could result in overestimation of accuracies due to pedigree structure. To reduce overestimation of accuracy, the (G)EBV were computed using the full data but with phenotypes on the validation animals and their progeny masked in the reduced dataset (GEBV_RED_). The accuracy of (G)EBV_RED_ of the validation animals was computed as: $\:acc=cov(GEB{V}_{RED},GEB{V}_{COM})/(1-\stackrel{-}{F}){\sigma\:}_{a}^{2}$ where $\:\stackrel{-}{F}$ is the average inbreeding coefficient in the validation population calculated using the tabular method [51]. Step two was repeated 5 times using 5 random validation sets and the final accuracy was averaged across the 5 replicates. To compare differences in accuracy between EBV and GEBV, the same 5 replicate validation set were used to compute pedigree-based EBV, with GEBV_COM_ and GEBV_RED_ substituted by GEBV_COM_ and EBV_RED_. The mean and standard error of the accuracies estimated for each of the five replicates were used to assess the differences between the accuracy of EBV_RED_ and GEBV_RED_ [52]. Additionally, a paired t-test was used to calculate the significance of the differences between EBV and EBV accuracy.

## Results and discussion

One of the challenges of complex traits measured across diverse environments is the consistency of records across evaluators and poorly defined traits. The IWDR database overcomes this challenge by providing for structured data entry of well-defined phenotypes scored by trained observers. This improves the likelihood that the same aspects of behavior are being assessed across service dogs from different organizations and breeders in different locations around the world. Of the 52 possible BCL items representing aspects of behavior traits, 17 traits on Labrador Retrievers bred for service work as guide dogs were analyzed in the current research, as listed in Table 1. An average information-restricted maximum likelihood method was used to estimate variance components and heritability for the 17 traits using pedigree and genomic information. Pedigree-based and genomic EBV were calculated, using a mix of pedigree, and genotype relationships for the latter. The accuracy of prediction with both methods was obtained using the LR Method [32], where the benchmark was the GEBV calculated with complete data. This approach is more consistent across populations subsets than predictive ability [52], which is calculated based on the correlation between the EBV and the phenotype corrected by fixed effects, and divided by the square root of heritability, and allows a straightforward implementation that fits the data from the current study.

### Heritability

**Fig. 1:**
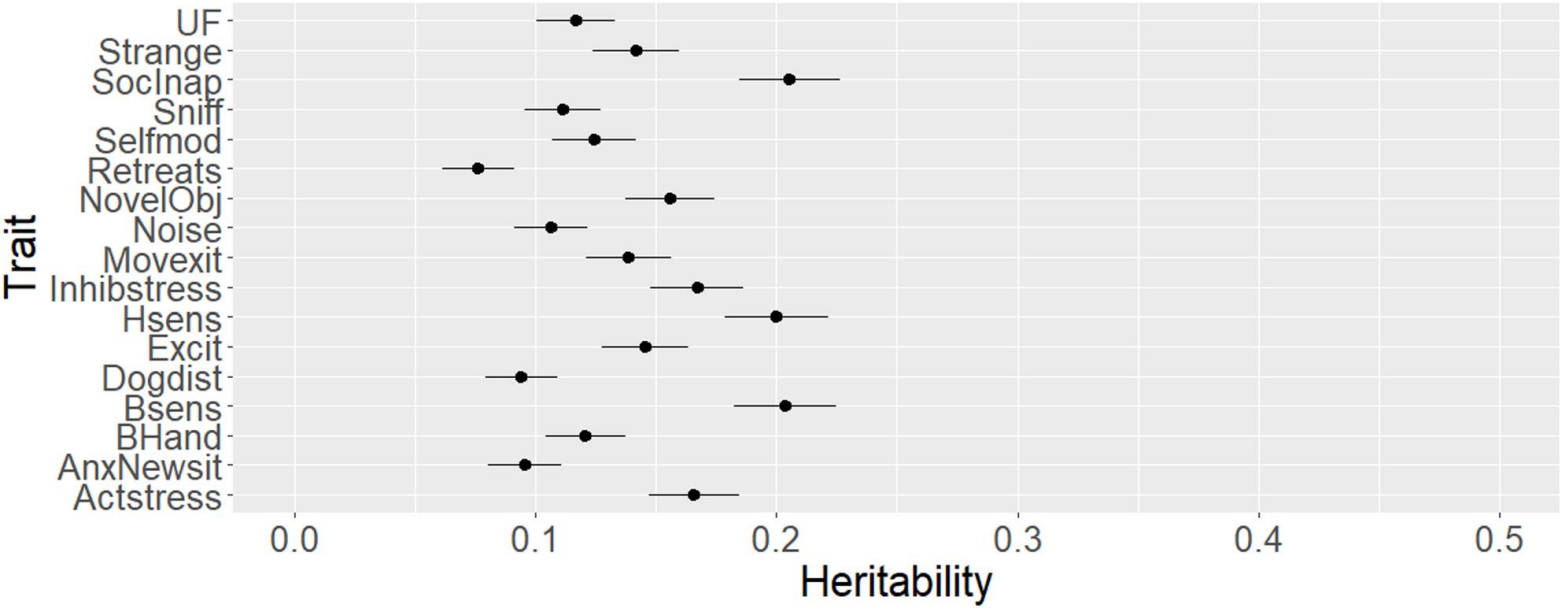
Estimates and standard errors of heritabilities of behavior traits. Heritability estimates for each of the 17 behavior traits in the behavior checklist. The bars represent the standard errors of the heritabilities

Figure 1 shows that the estimates (SE) of heritabilities were low to moderate, and ranged from 0.08 (0.01) (*Retreats*) to 0.21 (0.02) (*Socially inappropriate with people*). Differences between the variance components estimates with the inclusion of genomic data versus only pedigree were on the third decimal, and there were no differences in standard errors (results not shown). To the best of the authors’ knowledge, this is the first research paper to estimate heritabilities for standardized, robust behavior traits relevant to assistance dog success outcomes. Previous studies on behavior traits in dogs presented a wide range of heritabilities. For instance, Thorsrud et al. 2025 estimated heritabilities between 0.14 and 0.16 for high distractibility in Labrador Retrievers and other breeds, with no major difference in estimates between breeds or in a combined multi-breed analysis, but no SE were reported [53]. Wilsson and Sundgren [6] estimated heritabilities ranging from 0.05 to 0.35 in Labradors and 0.13 to 0.37 in German Shepherds, with SE of approximately 0.08 because phenotypes were available on only 797 animals. Additionally, Goddard and Beilharz [5] estimated heritabilities of behavior traits ranging from 0 to 0.58, which demonstrates a high variability in heritability estimates for behavior traits in dogs. Direct comparisons with the results from this work are not possible, since traits used in the cited literature were not included in the database used for this research. For instance, the ‘high distractibility’ used in [53] does not compare with the ‘distracted by dogs’ used in the present study due to differences in trait definition, although the goal of such measurements is likely the same. The length of time between this study and other references [5, 6] also complicates direct comparison. Further, phenotypes used in this study are based on standardized test protocols and each BCL item has definitions for each score point. Although scoring remains subjective, inter-rater reliability analyses conducted using an online scoring certification test indicates that people globally can be trained to score aspects of behavior consistently [29]. This increases the quality of the records and allows the inclusion of more dogs in the study. Yet, heritabilities are population-specific, and the estimates from the present study should be carefully evaluated in the context of highly trained and selected service dogs. Selection may reduce genetic variance, which may explain the low heritabilities for some traits, as several of the traits analyzed had already been under selection via pedigree-based EBVs for several years at the time of analysis. In addition, many behavioral traits are strongly impacted by the environment, especially during the critical period from birth through 4 months of age [54], which encompasses the period when the puppies are with their litter and the first two months with their volunteer puppy raiser. The quality and quantity of socialization provided by these volunteers vary considerably, especially for first-time puppy raisers [55]. The evaluators and the organizations in which the animals were scored could introduce variation to the observed phenotypes and are included in the contemporary group.

Estimates of heritability for behavior traits in other species vary according to the population and trait. In dairy cattle, heritability estimates for temperament range from 0.04 [56] and 0.07 [57] to 0.38 [58], indicating that values vary across populations and time periods. However, such a large range may also indicate a need for standardization of trait definitions and data collection [59]. A meta-analysis of temperament-related traits in beef and dairy cattle indicated that temperament is heritable [59]. In pigs, heritability estimates range from 0 (novel object tests and human approach tests) to 0.5 (total number of struggle attempts and total time spent struggling in the back test) [60]. Additionally, heritability of these traits in pigs tended to be higher in younger individuals, which may also indicate that acclimation and training have an impact on heritability estimates [60]. Our heritability estimates were mostly low, which could be a consequence of a naturally low genetic variance for traits in this population due to selection in service dogs over many generations preceding this study.

### Accuracy

**Fig. 2:**
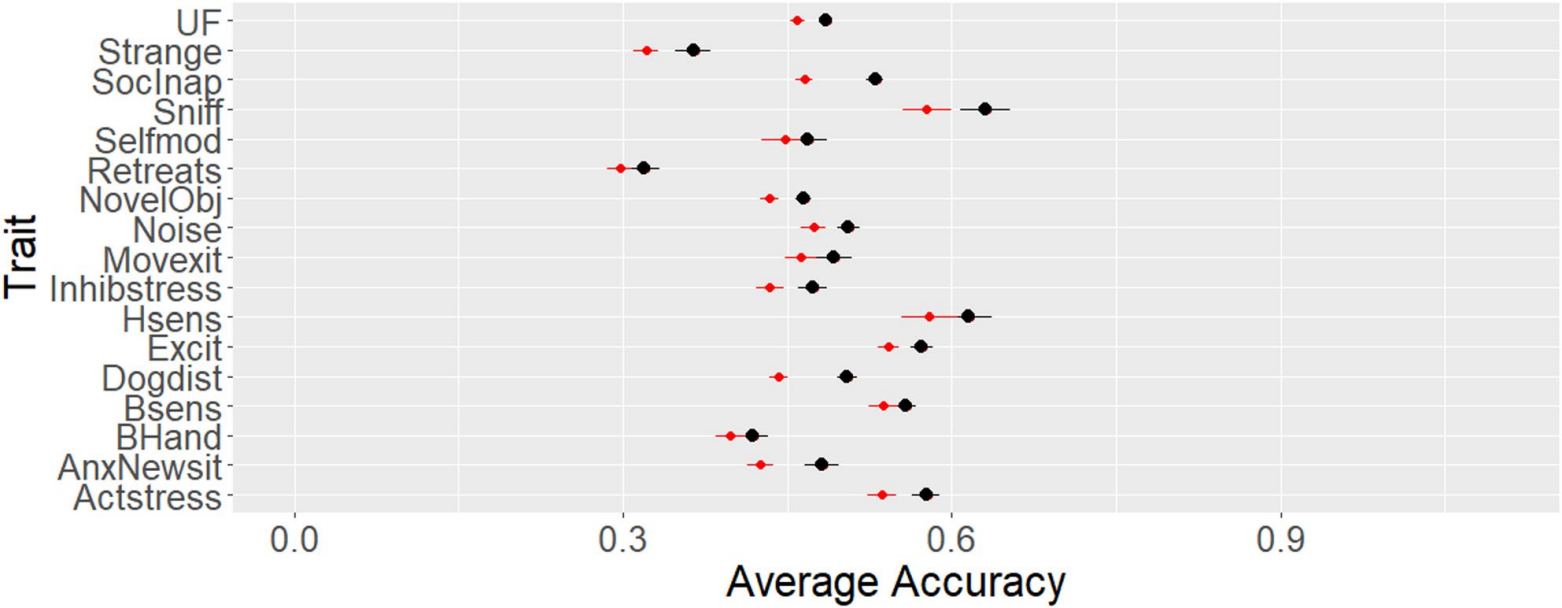
Average accuracy of genomic (GEBVs) and pedigree-based (EBVs) estimated breeding values across five validation replicates. Average accuracies of the GEBVs (in black) and EBVs (in red) for each of the 17 behavior traits in the behavior check. The horizontal bars represent the standard errors of the accuracy estimates

The accuracies obtained with the pedigree and the single-step evaluations are shown Fig. 2 and demonstrate a range from 0.29 to 0.63. Inclusion of genomic data increased accuracy in all 17 traits, ranging from 0.02 for *Body Handling* to 0.06 for *Socially inappropriate with people*. However, for six traits (*Self-Modulation*,* Retreats from known person*,* Excited by movement*,* Handle sensitivity*,* Body sensitivity*,* and Body Handling*), the standard errors of the accuracies with pedigree and single-step evaluations overlapped, and therefore, were not considered different. The paired t-test found no differences for those six traits and for *Distracted by scent* (*p* > 0.05); for all other trait differences, the differences in accuracy were significant (*p* < 0.05). It is well known that incorporating genomic information is expected to enhance the accuracy of EBV, as observed in several species [18, 19, 26, 27]. Non-significant increases in accuracy are usually not expected, but may be observed in some specific cases when problems arise from how traits are recorded [12], an insufficient number of markers [61] or when the number of records imposes a limitation in the validation approach [31]. The proportional increases in accuracy ranged from 4 to 14% of the accuracy of pedigree-based EBV, and for some traits were below expectations based on numbers from livestock breeding programs, which may be observed when the number of genotyped animals is not sufficient [63] or when scores for a trait are limited to few categories [24]. When evaluating the distribution of the phenotypes, most traits were skewed to the right, towards the most desired phenotype, which could contribute to lower increases in accuracy, especially when the sample size is limited, as observed in survival days in rainbow trout [61], which presented a similar phenotype distribution. Such differences in rainbow trout populations were corrected using weighted ssGBLUP approaches [61, 62], achieving high accuracies compared to the regular unweighted approach with censored and skewed phenotypes in rainbow trout [62]. However, those results could not be replicated in this study (results not shown) since weighted approaches require larger datasets for accurate predictions or the presence of major genes [63].

Accuracy of GEBV is a function of heritability and the numbers of genotyped animals, of animals in the training population, of independent chromosome segments, and of quantitative trait locis (QTLs) affecting the trait [30]. In the present study, we observed the expected positive relationship between heritability and accuracy of GEBV, with a significant (*p* < 0.05) correlation of 0.48. However, the correlation of differences in accuracy between EBV and GEBV with heritability was not significantly different from 0 (*p* > 0.05). Therefore, one of the reasons for differences in accuracy gains between traits is the number and distribution of QTLs, since the other terms are expected to be the same for all traits in this study. Therefore, the differences in accuracy between EBV and GEBV across the 17 traits could be explained by different genetic architectures, especially when heritabilities are similar. Finally, the fluctuation observed could be due to random noise and sampling errors in the validation approach.

The main factor influencing the validation of the GEBVs is the small number of genotyped animals available in this study. The limited number of genotyped animals imposes a limitation for accurate GEBV estimation, which is accentuated in more complex traits [63]. The number of genotyped animals that yield a plateau in accuracy is a function of the effective population size and the genome length in Morgans [64]. The number of independent chromosome segments (M_e_) in this study was 1277, based on the formula M_e_ = 2N_e_L/log(4N_e_L) [65], using values for the effective population size (N_e_) estimated between 114 and 122 [66, 67] and genome length (L) of 21 Morgan [68]. The N_e_ for Labrador used as guide dogs may be less than those estimates, and the actual Me should be lower. Therefore, there are not enough animals to accurately estimate the effects of all independent chromosome segments (ICS). This problem is exacerbated by the validation process, which masked a large proportion of the animals, in every round of validation, 100 of the 186 animals that had more than 10 progeny had their progeny and own records masked. Therefore, the limited number of records linked to genotypes in each round of validation only allowed estimation of the largest ICS [69]. This is a common issue in populations with large family size and a small number of genotyped animals [18, 19, 27] but should improve as more animals are genotyped and the number of individuals in the training population increases.

Weighted ssGBLUP decreased the accuracy for most traits (results not shown), as the estimate of the SNP variance for the chromosome segments with smaller effects shrank many SNP effects towards 0, causing a reduction in accuracy [70]. As an alternative, nonlinear weights [71] could control the shrinkage, but such methods depend on many parameters and are not advisable with a small dataset. The adoption of weighted ssGBLUP in commercial applications is not common, and gains are limited in most cases [15–17], and the adoption of such approaches for multiple traits requires a complex implementation. Thus, the use of weighted approaches is not recommended at early stages of development of a genomic selection program, unless a major gene or an oligogenic genetic architecture is identified [62].

Selecting animals randomly for the validation is not ideal, as it may include siblings in the training and validation population, which would inflate the accuracy [25]. Additionally, random cross-validation does not account for changes in allele frequencies across generations, which may bias the validation accuracy [32]. The effects of selection for behavioral traits in the validation results are not a significant concern at this time, as selection based on EBVs has occurred for only a handful of behavioral traits. Additionally, selection based on phenotypes is not expected to have a large impact due to the low heritability magnitude observed in this population. EBVs for harness and body sensitivity were introduced in an index in 2018, and since 2019, noise and object fear. Additionally, ssGBLUP accounts for the pre-selection of animals, which controls biases in genomic predictions [72]. Nevertheless, the validation approach must be changed to forward-in-time validation when the genomic selection program is fully implemented and more genotypes are available, such that validation mimics the selection of young animals. An intermediate approach between a random and a forward-in-time cross-validation is using k-means to select unrelated individuals [25], which will be possible when more genotypes are available.

Despite the limitations imposed by the small number of genotyped animals, the results are promising. The number of genotyped animals is expected to increase once GEBVs are implemented in regular evaluations and more reliable EBV are calculated, a trend observed in Dairy Cattle [23] and other livestock populations. Therefore, once more genomic data is available, differences between EBV and GEBV accuracies are also expected to increase. Additionally, genotyping costs are expected to decrease with increasing enrollment, which is expected once the breeding program adopts genomic selection. The use of commercial SNP chips instead of WGS may also contribute to lower costs and faster turnaround, without compromising the quality of genotypes [73]. Additionally, the ideal marker density for this population must be studied. However, a canine panel must be useful across multiple breeds, so a higher-density chip is beneficial, as it should contain enough polymorphic SNPs for many breeds. Finally, genotyping costs for young animals can be further decreased by adopting low-density panels that can be imputed to the higher-density chips used in this study [74, 75].

## Conclusions

Estimates of heritabilities for behavior traits in this population of Labrador Retrievers ranged from 0.07 to 0.20. Including genomic information improved the accuracy of EBV compared to pedigree-based EBV for all traits, highlighting the potential of genomic selection for improving behavioral phenotypes essential to guide dog success. However, the gains in accuracy from genomic selection were less than those observed in other species due to the limited number of genotyped animals. A practical and impactful next step is to expand genotyping efforts, particularly among breeding animals with many phenotyped progeny, which is expected to enhance the reliability and effectiveness of genomic selection. Taken together, these results provide an initial proof of concept for genomic prediction for behavioral traits in guide dogs and provide a foundation for implementing genomic selection to support working dog success outcomes.

## Data Availability

The data used in this research belongs to Guiding Eyes for the Blind or Guide Dogs New South Wales and must remain confidential. IWDR-affiliated authors should be contacted to share data, and they will jointly decide if data will be shared based on potential use by the other party.
